# Efficacy and safety of moxibustion for menstrual irregularities: A protocol for systematic review and meta-analysis

**DOI:** 10.1097/MD.0000000000025281

**Published:** 2021-04-09

**Authors:** Li Liu, Min Liu, Meinian Liu, Yufang Gui, Lei Sun, Xiaoyun Zuo

**Affiliations:** a Jiangxi Province Hospital of Integrated Chinese and Western Medicine; b The Affiliated Hospital of Jiangxi University of Traditional Chinese Medicine, Nanchang, China.

**Keywords:** menstrual irregularities, moxibustion, protocol, randomized controlled trial

## Abstract

**Background:**

Menstrual irregularities (MI) is 1 of the most common clinical gynaecological diseases, with abnormal menstrual cycles, abnormal bleeding, and abdominal pain before or during menstruation as the main clinical manifestations. In modern medicine, abnormalities in the function of the pituitary gland, hypothalamus, and ovaries can affect menstruation. Currently, hormone levels in the body are mostly regulated by hormonal drugs, but these drugs can lead to hormonal imbalance, which can lead to adverse reactions. Many clinical studies have reported that moxibustion has a good effect on MI treatment, but there is no relevant systematic review. So the purpose of this study is to evaluate the effectiveness and safety of moxibustion in treating MI.

**Methods:**

The following 8 electronic databases will be searched, including PubMed, Embase, the Cochrane Library, China National Knowledge Infrastructure, Web of Science, Chinese Scientific Journal Database, Wanfang Database, and Chinese Biomedical Literatures Database from their inception to 1 December 2020 without any restrictions. Researchers retrieve the literature and extracted the data, evaluation of research methods, quality of literature. The outcomes will include total effective rate, incidence of any adverse events. We use the Cochrane Risk of a bias assessment tool to evaluate methodological qualities. Data synthesis will be completed by RevMan 5.3.0.

**Results:**

We will show the results of this study in a peer-reviewed journal.

**Conclusions:**

This meta-analysis will provide reliable evidence for treatment of menstrual irregularities.

**INPLASY registration number:**

INPLASY2020120042

## 1 Introduction

Menstrual irregularities (MI) is common gynaecological disorders that manifest as abnormalities in the menstrual cycle or bleeding and may be accompanied by abdominal pain before and during menstruation and systemic symptoms. The cause may be organic lesions or malfunction.^[1]^ The occurrence of this disease is related to a variety of factors, such as emotional abnormalities, cold stimulation, dieting, etc.^[2]^ 75% of adult women are affected by irregular menstruation and one of the reasons for their frequent visits to the hospital.^[3]^ There are various clinical manifestations of menstrual irregularities, mainly irregular uterine bleeding, dysfunctional uterine bleeding, amenorrhea, menopause, etc.^[4,5]^ They often feel fatigued, bloating, anger and moodiness, which seriously affects their study and life.^[6,7,8]^

The current primary treatment includes hemostasis and correction of anaemia, and cycle therapy with estrogen and progestin alone or in combination.^[9]^ a high success rate has been shown in the treatment of MI with estrogen, However, benefits may be short-lived, and adverse effects have been reported.Moxibustion, as a complementary and alternative therapy, has been developed in China for thousands of years. Moxibustion has a relatively good effect in the treatment of MI. A large number of clinical studies on the treatment of MI with moxibustion have been reported, but there is no relevant systematic review. So this research aims to systematically and comprehensively evaluate the safety and efficacy of moxibustion in the treatment of MI.

## 2 Methods

### 2.1 Study registration

This protocol was registered with the International Platform of Registered Systematic Review and Meta-Analysis Protocols (INPLASY) on 04 December 2020 (registration number INPLASY2020120042). It could be obtained from https://inplasy.com/inplasy-2020–12–0042. This report will be conducted based on the preferred reporting items for systematic reviews and meta-analyses protocols (PRISMA) statement guidelines.^[10]^

### 2.2 Inclusion criteria

#### 2.2.1 Study type.

All randomized controlled trials (RCTs) and quasi-RCTs study on moxibustion in the treatment of MI will be included.

#### 2.2.2 Participants.

All patients included in the study were diagnosed with MI, regardless of age, gender, race.

#### 2.2.3 Type of intervention

The trail group uses moxibustion or combination therapy with other treatments. The moxibustion method, acupoint selection, and needles are not limited; the control group uses the progesterone, and other adjuvant treatments can be appropriately added. The trail group and the control group are not limited in terms of medication, dosage, and treatment course.

#### 2.2.4 Type of outcome measures

##### 2.2.4.1 Primary outcomes

Primary outcome measures will include:

1. The effective rate.
2. Symptom improvement.

##### 2.2.4.2 Secondary outcomes.

Secondary outcome measures will include:

1. Incidence of any adverse events.

### 2.3 Exclusion criteria

The following exclusion criteria are presented:

1. The same study or duplicated publications.
2. Case report.
3. Theoretical or basic research.
4. Unable to get available data through various means.

### 2.4 Search strategy

The following 8 electronic databases will be searched, including PubMed, Embase, the Cochrane Library, China National Knowledge Infrastructure (CNKI), Web of Science, Chinese Scientific Journal Database(VIP), Wanfang Database, and Chinese Biomedical Literatures Database (CBM) from their inception to November 2020 without any restrictions. We strictly follow the PRISMA^[11]^ statement, The research only includes human subjects, The main search terms: “moxibustion,” “menstrual irregularities,” and “randomized controlled trial.” will be included. We use similar search strategies for all electronic databases. We will also search for eligible trial, which is unpublished or ongoing. The search strategy for PubMed is shown in Table 1.

**Table 1:** The search strategy used in the PubMed database.

Search	Query
#1	“Menstrual Irregularities”[MeSH Terms]
#2	((((((Disturbance, Menstruation[Title/Abstract]) OR (Disturbances, Menstruation[Title/Abstract])) OR (Menstruation Disturbance[Title/Abstract])) OR (Menstruation Disorders[Title/Abstract])) OR (Disorder, Menstruation[Title/Abstract])) OR (Disorders, Menstruation[Title/Abstract])) OR (Menstruation Disorder[Title/Abstract])
#3	#1 or #2
#4	“Moxibustion”[Mesh]
#5	(Moxabustion[Title/Abstract]) OR (Acupuncture[Title/Abstract])
#6	#4 or #5
#7	randomized controlled trial[Publication Type] OR randomized[Title/Abstract] OR placebo[Title/Abstract]
#8	#3 and #6 and #7

### 2.5 Study selection

**Figure 1:**
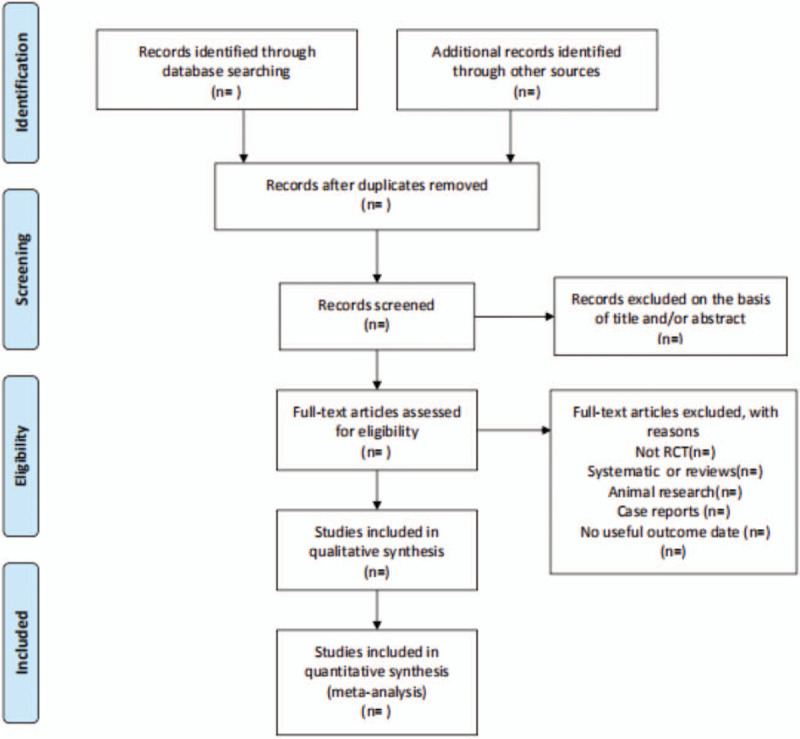
Flowchart of literature selection.

All literature retrieved from electronic databases will be imported into NoteExpress 3.2.0 software for category management, excluding double-checked and published literature. All researchers will discuss and define the selection criteria before selecting the literature. Two reviewers will independently assess the retrieved studies against the inclusion criteria. In the initial selection of studies, only titles and abstracts will be reviewed to exclude inappropriate publications. Studies that do not match will be removed to the trash folder in the software. Reasons for exclusion will be recorded as an Excel dataset. The next step will be to further evaluate the included studies by reading the full text. Two reviewers will check the reference list to identify trials that may have been missed. Two reviewers will cross-check the screening results. The reviewers, if they disagree, will resolve their disagreement by consensus. Any disagreement will be resolved by discussion between the 2 authors and the third author, arbitration, if necessary. The above process will be presented in the form of a PRISMA flowchart. (Fig. 1)

### 2.6 Data extraction and management

Data will be extracted independently from a pre-established data extraction table. The following basic information will be extracted: author, year, country, sample size, mean age, randomized method, intervention, control measures, follow-up time, outcome measures, and adverse effects. 3 reviewers will cross-check the results. They are (Li Liu, Min Liu, and Meinian Liu). If there is any disagreement, it will be negotiated or arbitrated by the fourth investigator (Lei Sun). Besides, the intention-to-treat analysis of this study will be used to address missing data. Data. NoteExpress 3.2.0 and Excel 2007 software will be applied. To extract the eligible data. When vital data in the study are incomplete or missing, we will have the option to contact the first author or the corresponding author to obtain the data by phone or email.

### 2.7 Assessment of risk of bias in included studies

The risk of bias in the included studies will be assessed by two independent reviewers (Li Liu and Min Liu) using the assessment tool of Cochrane Reviewer's Handbook 5.0.24.^[12]^ We will assess the risk of bias in the following areas: allocation sequence generation, allocation sequence concealment, blinding of personnel and outcome assessors, incomplete information. Outcome data, selective reporting of outcomes, and other sources of data. Bias. The assessment will be divided into 3 levels: low risk, high risk, and Unclear Risk. Ambiguous items in the study will be queried by Contact the appropriate author for details. Any disagreement will be resolved through discussion with the third reviewer (Lei Sun).

### 2.8 Data synthesis

#### 2.8.1 Measures of treatment effect

We will use RevManV.5.3.0 software for data analysis and quantitative data synthesis. Dichotomous data will be analyzed using risk ratios with 95% confidence intervals. For continuous outcomes, we will analyze using the mean difference or standardized mean difference with 95% confidence intervals,^[13]^ when fewer than two studies are included for each outcome measure, only descriptive analyses will be performed to summarize the results.

#### 2.8.2 Heterogeneity analysis

Statistical heterogeneity between included studies will be assessed strictly according to the criteria (*P* > .1 and *I*^2^ < 50%) and displayed as a forest plot. When *P* > .1 and *I*^*2*^ < 50%, lower heterogeneity was analyzed using a fixed-effects model; when *P* < .1 and *I*^2^ > 50%, higher heterogeneity was analyzed using a random-effects model. When the included studies’ heterogeneity is significant, we will choose subgroup analysis or sensitivity analysis to search for possible sources clinically and methodologically.

#### 2.8.3 Subgroup analysis

If sufficient data are available, we will conduct subgroup analyses based on the following themes: age, duration of treatment, study quality, type of intervention in the control or study group, and so on.

#### 2.8.4 Sensitivity analysis

Sensitivity analysis will be used to validate the robustness of the review findings. We will consider several decision points in the systematic review process to implement the sensitivity review, such as sample size, missing data results, and methodological quality. Besides, the analysis will be repeated after excluding studies with low methodological quality.

#### 2.8.5 Publication bias

When the number of eligible RCTs is ≧ 10,^[14]^ publication bias will be detected using a funnel plot developed by Egger test.

#### 2.8.6 Grading the quality of evidence.

A summary table of findings will be generated and included in the final report. Three investigators will assess the quality of each selected study through a tiered proposal assessment, development, and evaluation methodology. The following areas will be assessed: risk of bias, consistency, directness, precision, publication bias, and additional scores. There will be four levels of assessment: high, medium, low, and very low quality.^[15]^

#### 2.8.7 Ethics and dissemination

Ethical approval is not required as data from individual patients are not included, and there are no privacy implications. We will disseminate the results of this systematic review by publishing the manuscript in a peer-reviewed journal or presenting the relevant conference findings.

## 3 Discussion

The causes of menstrual irregularities are varied and affect different patients in different ways. Menstrual irregularities can lead to various serious problems and in severe cases can lead to endometrial cancer and infertility. The treatment of irregular menstruation in the clinical field mainly includes Western medical treatment and Chinese acupuncture treatment. Western medical treatment is in the form of hormone therapy, and progesterone is commonly used, although the treatment effect is better, patients are prone to adverse problems, which affect the final result of treatment. In recent years, scholars have increased the clinical research on menstrual disorders, and the results of related practical research show that moxibustion treatment based on the idea of dialectical treatment in Chinese medicine can play an excellent role in the treatment of menstrual disorders. In recent years, scholars have increased clinical research on menstrual disorders. However, there is a lack of systematic reviews and meta-analyses of moxibustion treatment for MI. Therefore, we intend to demonstrate the efficacy and safety of moxibustion for MI through this systematic review and meta-analysis. Finally, we hope that the results of this review will provide clinicians with more reliable, evidence-based evidence for the treatment of MI.

## Author contributions

**Conceptualization:** Li Liu, Min Liu.

**Data curation:** Li Liu, Min Liu, and Meinian Liu.

**Formal analysis:** Li Liu, Min Liu, and Meinian Liu.

**Investigation:** Lei Sun, Li Liu.

**Methodology:** Yufang Gui, Xiaoyun Zuo.

**Software:** Min Liu, Meinian Liu.

**Supervision:** Meinian Liu, Lei Sun.

**Writing – original draft:** Li Liu, Min Liu, and Meinian Liu.

**Writing – review & editing:** Lei Sun, Yufang Gui, Xiaoyun Zuo.
